# VIP-DB: A Comprehensive Database of Virus–Insect–Plant Relationships

**DOI:** 10.3390/v18060679

**Published:** 2026-06-18

**Authors:** Tao Deng, Dandan Liu, Xinghui Zhu, Hongyan Zhang, Zheng Zhang

**Affiliations:** 1 College of Information and Intelligence, Hunan Agricultural University, Changsha 410128, China; 2089644379@stu.hunau.edu.cn (T.D.); zhuxh@hunau.edu.cn (X.Z.); 2 Department of Interdisciplinary Science and Smart Seed Industry Equipment, Yuelushan Laboratory, Changsha 410128, China; 3 College of Plant Protection, Hunan Agricultural University, Changsha 410128, China

**Keywords:** plant viruses, insects, virus transmission modes

## Abstract

Insect-mediated transmission is central to the epidemiology of plant viruses and has major implications for global food security and agricultural production. Although several resources have compiled information on plant virus transmission, evidence-traceable integration of virus–insect vector–host plant relationships remains limited. Here, we developed the Virus–Insect–Plant Database (VIP-DB), an evidence-guided database that links literature-derived virus–insect transmission records, host plant information, transmission mode annotations, taxonomic information, and traceable literature evidence. VIP-DB compiles 583 virus–insect transmission relationships, 855 virus–plant relationships with non-missing host plant information, and 1375 integrated virus–insect–plant records. Among these records, 120 lack host plant information and 51 lack transmission mode annotation. VIP-DB provides a curated and searchable resource for querying documented plant virus, insect vector, host plant, and transmission mode information. This database offers an evidence-traceable framework for comparative analyses of plant virus transmission relationships and supports future studies in plant virology, vector ecology, and disease management.

## 1. Introduction

Plant diseases threaten global food security, and viruses alone account for roughly half of these losses [1,2,3]. Of all described plant viruses, about 80% are transmitted by insect vectors, especially *Hemiptera*, that mediate the majority of viral spreads and cause the greatest economic losses [4]. Transmission relationships between viruses and insect vectors can be specific and often multiplex: a single virus may exploit several vectors, and a single vector may carry multiple viruses. Tomato spotted wilt virus (TSWV) exemplifies the former pattern, being transmitted by both western flower thrips and tobacco thrips [5,6,7]. In contrast, the green peach aphid demonstrates the latter, capable of vectoring potato virus Y, faba bean necrotic stunt virus, and potato leafroll virus [8,9,10]. Despite their economic importance, resources that integrate virus–insect vector relationships with host plant records, transmission mode annotation, and traceable literature evidence remain limited. Existing classification frameworks and datasets further limit our understanding of these complex relationships.

In the 1960s, Hull et al. classified plant virus–vector relationships into three categories: non-persistent, persistent circulative, and persistent propagative [11]. Subsequent refinements subdivided these into four transmission modes: non-persistent, semi-persistent, persistent non-propagative, and persistent propagative—now widely used in the field [12,13]. However, these classifications remain insufficient for integration into comprehensive transmission network frameworks. While previous studies have revealed that major insect vector groups exhibit characteristic transmission modes (representatives of the family *Aphididae* predominantly mediate non-persistent virus transmission, whereas whitefly vectors primarily mediate persistent non-propagative transmission), these conclusions are mainly based on empirical descriptions, whereas cross-family curation of Virus–Insect–Plant records and sequence-based comparison remain limited [14].

Existing resources have provided important foundations for plant virus transmission research, but they differ from VIP-DB in focus, data structure, and downstream functions. Wu et al. (2024) analyzed 24 plant viruses to map vector interactions [15], while Wang et al. (2023) developed the *Geminiviridae*-Plant-Insect Database (GPI-base) covering only four insect vectors and lacking transmission mode annotations [16]. More recently, Peters et al. (2024) reported the Plant Virus Transmissions Database, which compiles transmission modes and vectors for more than 1600 plant viruses based on over 3500 publication records spanning approximately 100 years [17]. This database provides an important broad resource for plant virus transmission information. In contrast, VIP-DB was designed to complement these resources by focusing specifically on structured virus–insect–plant relationships. VIP-DB integrates manually curated virus–insect transmission records, host plant information, transmission mode annotations, taxonomic information, and traceable literature evidence. Therefore, VIP-DB provides a curated database for querying plant virus–vector–host relationships and for supporting comparative analyses of documented insect-mediated transmission patterns.

Because viral evolution continually reshapes host range and transmission risk, comprehensive data are essential for anticipating new outbreaks [18]. We therefore constructed the Virus–Insect–Plant Database (VIP-DB), integrating transmission relationships for 447 plant viruses, 380 host species, and 100 insect vectors. These records provide a basis for describing documented patterns of specificity and diversity in plant virus transmission and for supporting future studies in plant virology, vector ecology, and disease management.

## 2. Materials and Methods

### 2.1. Data Collection

Virus–insect transmission data: A literature search was conducted on 22 August 2023, using the keywords “plant virus” and “insect” in PubMed. A total of 1231 articles were retrieved, including 904 with full-text access and 327 with abstract-only availability. Additionally, data on *Geminiviridae*–insect transmission relationships were sourced from the GPI-base database (http://gpi.geminiviridae.com/, accessed on 31 January 2024). Literature abstracts and data were mined using the Dara website (https://chat.portal.so/, accessed on 31 January 2024), an online platform powered by GPT-4 Turbo.

Virus–plant infection data: Detailed virus–plant infection data were retrieved from the Virus–Host DB (https://www.genome.jp/virushostdb/, accessed on 15 June 2023).

Additional data: To build the dataset for the virus transmission pattern prediction model, 323 viral protein and 384 genome sequences were downloaded from the NCBI database (https://ftp.ncbi.nih.gov/refseq/release/viral/, accessed on 26 February 2024). The Baltimore classification of plant viruses was queried from ViralZone (https://viralzone.expasy.org/, accessed on 21 March 2024).

### 2.2. Extraction of Virus–Insect Transmission Relationships

To efficiently extract information on virus–insect transmission relationships and transmission modes from the literature, we employed Dara website, an online platform powered by a GPT-based large language model. Literature abstracts were uploaded individually, and key information was extracted using the GPT-4 Turbo model. During this process, specific cue words and prompts were used to guide the extraction of relevant data.

First, we used the prompt “Is this a review article?” for confirmation. If the answer was “Yes”, we checked whether the article contained a table with virus–insect transmission relationships. If such a table was present, its contents were manually copied into Dara website, and the prompt “According to the table, please list the relationship between the virus, insects, virus protein–insect protein interaction, and the transmission types between virus and insect” was used to extract the information.

Second, if the answer was “No”, we used the prompt “Please give me four columns of table information based on this document, including the virus, the insect hosts that transmit the virus, the transmission mode of the virus, and the evidence corresponding to the original text. If there are no values, fill in ‘null’” to extract the information.

Finally, Dara website generated a table including the virus name, insect species, transmission mode, and associated DOI. If the table was not generated, regeneration was attempted.

### 2.3. Data Validation

To improve the credibility of the information extraction, we manually verified the data, focusing on whether the evidence supports the virus–insect transmission relationships and transmission modes. We defined two reference columns in Supplementary Table S1 for evidence tracing: Column DOI for Validation Virus–Insect Relationships contains literature references confirming the existence of virus transmission by insect vectors, and Column DOI for Validation Virus Transmission Mode includes literature references verifying the corresponding virus transmission mode. If the evidence was unclear or inconsistent with the literature in the corresponding DOI columns, we supplemented relevant references through platforms like Google Scholar to ensure the accuracy and reliability of the transmission relationships and mode annotations.

To ensure the completeness of the virus–insect transmission data, we downloaded virus–insect transmission data for bipartite viruses from the GPI-base database. Due to missing data in GPI-base, we manually filtered and supplemented virus data with insect vector information, incorporating the persistent-propagative transmission mode for bipartite viruses into the dataset.

### 2.4. Classification of Transmission Relationship

To ensure consistency and uniformity in the database, we standardized the names of viruses and insects according to NCBI naming conventions. For cases where the literature described transmission as “Persistent, circulative” without distinguishing between “propagative” or “non-propagative”, we introduced the “Persistent, circulative” category (Supplementary Table S1) to more accurately classify persistent transmission viruses. We also obtained virus and insect classification information from the NCBI database and integrated it into the final database.

Ultimately, an integrated dataset comprising 1375 virus–insect–plant records was generated based on the integration of 583 virus–insect transmission relationships and 855 virus–plant infection relationships with non-missing host plant information (Supplementary Table S1). Of these 1375 records, 120 lack host plant information and 51 lack transmission mode annotation.

### 2.5. Virus–Insect–Plant DB Platform Design

The platform’s backend was developed using Java version 17 (Oracle Corporation, Redwood Shores, CA, USA; https://www.oracle.com/java/, accessed on 14 June 2026), and was built with the Spring Boot framework version 3.1.5 (Broadcom Inc., Palo Alto, CA, USA; https://spring.io/projects/spring-boot, accessed on 14 June 2026). Data management was performed using MySQL version 8.0.33 (Oracle Corporation, Redwood Shores, CA, USA; https://www.mysql.com/), and efficient data operations were implemented using MyBatis-Plus version 3.5.7 (Baomidou Development Team, China; https://baomidou.com/). Lombok version 1.18.28 (Project Lombok Authors; https://projectlombok.org/), was used to simplify boilerplate code, while Hutool version 5.8.11 (Guizhou Bugou Technology Co., Ltd., Guiyang, China; https://hutool.cn/), provided standard utility classes. The platform’s API documentation was automatically generated using SpringFox version 3.0.0 (SpringFox Development Team; https://springfox.github.io/springfox/, accessed on 14 June 2026), and system testing was performed using the Spring Boot Test module.

### 2.6. Statistical Analysis

All data processing in this study was performed using Python version 3.8.12 (Python Software Foundation, Beaverton, OR, USA; https://www.python.org/). Graphs were created using R version 4.4.0 (R Foundation for Statistical Computing, Vienna, Austria; https://www.r-project.org/), with the pheatmap package version 1.0.12 (maintained by Raivo Kolde; https://cran.r-project.org/package=pheatmap, accessed on 14 June 2026). Diagrams were drawn using the draw.io online tool (draw.io Ltd., Northampton, UK; https://www.drawio.com/).

## 3. Results

### 3.1. Analysis of Virus–Insect Transmission Relationships

To investigate transmission relationships between insects and plant viruses, we constructed VIP-DB, a comprehensive and systematically curated resource, by systematically extracting data from related literature and integrating existing databases. The VIP-DB currently includes 583 virus–insect transmission relationships across 447 plant viruses (30 families, excluding an “Unclassified” category) and 100 insect vector species (6 orders, 16 families). Among the 1375 integrated records, 1255 include host plant information, spanning 380 plant species, whereas 120 lack host plant information and 51 lack transmission mode annotation (Supplementary Table S1). The primary focus of the database is on documenting insect-mediated transmission pathways and modes, while plant host information serves as contextual metadata for each transmission relationship.

**Figure 1:**
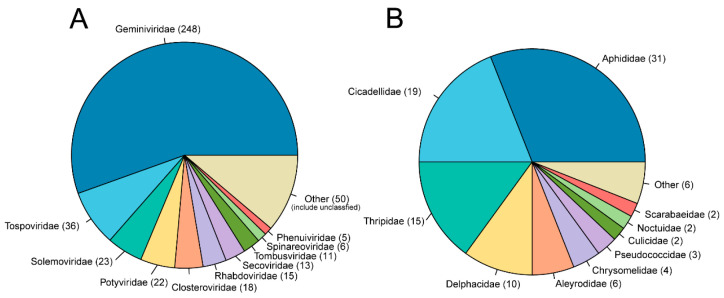
Distribution of the number of plant viruses and insect species. (**A**) Distribution of plant viruses grouped by viral family. (**B**) Distribution of insect vectors grouped by insect family. Note: Numbers in parentheses indicate the number of species in each family.

To comprehensively analyze the distribution of the data compiled in the database, we first analyzed the distribution of plant viruses and insect families within the collected dataset. Among virus families, *Geminiviridae* contained the highest number of viruses, totaling 248 species (55.48% of all viruses). This number was significantly higher than the second-ranked family, *Tospoviridae*, which contained 36 virus species (Figure 1A). Among insect families, *Aphididae* contained the largest number of insect species (31 species), followed by *Cicadellidae* with 19 species (Figure 1B). In terms of plant hosts, among the 380 plant species recorded in the database, 256 species (67.37%) were recorded as hosts of only 1 virus, and 343 species (90.26%) were recorded as hosts of fewer than 5 viruses, suggesting that most plant species in the current dataset are associated with a limited number of viruses. Only 4 plant species were recorded as hosts of 20 or more viruses: *Solanum lycopersicum* was infected by 35 virus species, followed by *Nicotiana benthamiana* (31 virus species), *Phaseolus vulgaris* and *Nicotiana tabacum* (20 virus species each). These results indicate that virus infection exhibits notable host specificity, with the majority of plant species serving as hosts for only a few viruses, while a small number of plant species act as major virus reservoirs.

**Figure 2:**
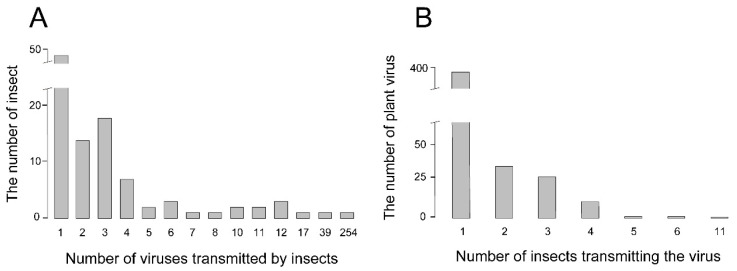
Quantitative distribution of plant virus–insect transmission relationships. (**A**) Number of plant viruses transmitted by each insect. (**B**) Number of insects transmitting each plant virus.

Based on the collected virus–insect transmission relationships, the number of plant viruses transmitted by insects in the database was further analyzed. As shown in Figure 2A, 76.00% of all insects were those transmitting 3 or fewer plant viruses. Specifically, 45 insect species transmitted only 1 virus, indicating a high level of specialization. Additionally, 14 species transmitted 2 viruses, and 17 species transmitted 3 viruses. Insects capable of transmitting more than 3 plant viruses represented a relatively small proportion. For example, *Bemisia tabaci* transmitted the highest number of plant viruses (254 viruses), among which 234 viruses belonged to the family *Geminiviridae*, accounting for 92.13% of its total transmitted viruses (Supplementary Table S1). *Myzus persicae* is an insect vector for 39 virus species, most notably from the family *Solemoviridae* with 10 virus species (such as pea enation mosaic virus 1) and the family *Potyviridae* with 15 virus species (including the potato virus Y), which together represent 64.10% of the total virus species transmitted by this insect (Supplementary Table S1). These data are consistent with well-established evidence that *Bemisia tabaci* is a major vector of *Geminiviridae* family and that *Myzus persicae* plays a prominent role in the transmission of viruses belonging to the *Solemoviridae* and *Potyviridae* families.

As shown in Figure 2B, 378 viruses (84.56%) were transmitted by only 1 insect species, while 30 viruses were transmitted by 2 insect species, and the remaining viruses were transmitted by more than 2 insect species. These data indicate that most viruses rely on a single insect vector, with only a few viruses transmitted by multiple insect species, further demonstrating insect specialization in virus transmission. Among these, barley yellow dwarf virus (BYDV) and rice tungro spherical virus (RTSV) were transmitted by 5 insect species, while pea enation mosaic virus 1 (PEMV) and rice tungro bacilliform virus (RTBV) were transmitted by 6 insect species. Notably, TSWV exhibited a more complex transmission mode, being transmitted persistently by 11 insect species (Supplementary Table S1). This case is consistent with previous studies, indicating that certain viruses, such as TSWV, possess relatively complex transmission mechanisms.

### 3.2. Distribution Characteristics of Viral Transmission Modes

**Figure 3:**
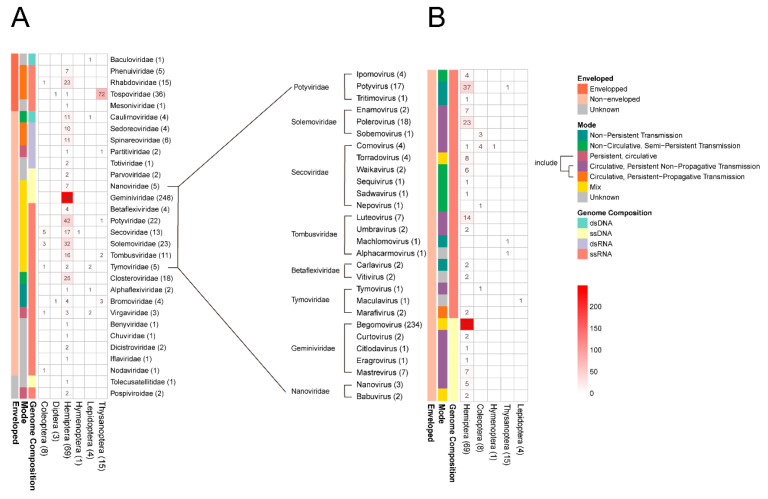
Relationships between plant viruses and insect orders. (**A**) Distribution of virus families transmitted by insect orders. (**B**) Distribution of virus genera transmitted by insect orders. Note: Numbers in the central matrix indicate the number of virus–insect relationships in each virus family or genus across insect orders. Numbers in parentheses after virus family or genus names indicate the number of virus species, and numbers in parentheses after insect order names indicate the number of insect species. Only plant viruses with defined family assignments in the NCBI database were included; 3 viruses currently classified as “Unclassified” were excluded. For detailed classification information of the viruses used in this analysis, refer to Supplementary Table S1.

Then, the relationships between key virus families and insect orders were analyzed, and the biological characteristics and transmission modes of the viruses were characterized. Within the family *Geminiviridae*, the genus *Begomovirus* included 234 virus species, representing approximately 94.35% of viruses in this family (Figure 3B). At the species level, all 248 *Geminiviridae* virus species were transmitted by *Hemiptera*, highlighting the critical role of *Hemiptera* as vectors of *Geminiviridae*. *Hemiptera* was the most diverse insect order transmitting plant viruses, associated with 28 virus families out of 30 total virus families, accounting for 93.33%. *Aphididae* is the most speciose family within the order *Hemiptera*, with 31 insect species out of 69 total *Hemiptera* species, accounting for 44.93%. As previously mentioned, *Bemisia tabaci*, which transmits the greatest number of plant viruses, also belongs to *Hemiptera*. *Thysanoptera* transmitted 42 virus species across 5 virus families, of which 36 belonged to the family *Tospoviridae*, representing 85.71% of viruses transmitted by *Thysanoptera*. These data recapitulate the well-established relationship between *Thysanoptera* and viruses of the family *Tospoviridae* (Figure 3A).

To examine the potential association between plant virus transmission modes and virus envelope status, we further analyzed the envelope annotations of the viruses included in VIP-DB. In the current dataset, non-persistent transmission was not typical of viruses annotated as having envelopes, whereas viruses without envelopes were associated with multiple transmission modes, including persistent and non-persistent transmission. At the family level, viruses belonging to 23 families (76.67%) were annotated as lacking envelopes, with many of these viruses assigned to *Geminiviridae* and *Potyviridae*. In contrast, viruses from 5 families (16.67%) were annotated as having envelopes, mainly including viruses assigned to *Rhabdoviridae* and *Tospoviridae* (Figure 3A). The envelope status of viruses from several families remained undetermined.

Regarding the distribution of plant virus transmission modes, persistent transmission was dominant, observed in 364 viruses out of 447 total viruses, accounting for 81.43% of all viruses. Specifically, viruses in the family *Geminiviridae* exhibited mixed persistent transmission modes, including persistent and persistent non-propagative types. Viruses in the family *Potyviridae* showed mixed transmission modes (non-persistent and semi-persistent), collectively categorized as non-persistent (Figure 3B). Most annotated viruses from the family Tospoviridae exhibited a persistent-propagative transmission mode (Figure 3A). Among the virus genera analyzed, only *Torradovirus* exhibited both persistent and non-persistent transmission modes, while the remaining genera demonstrated consistent transmission modes. This observation supports the general assumption that plant virus genera tend to exhibit consistent transmission modes (Supplementary Table S1).

According to the Baltimore classification, the plant viruses included in VIP-DB comprised 256 ssDNA virus species (57.27% of the total), which were assigned to 4 virus families, with most belonging to *Geminiviridae* (248 species). The dataset also included 170 ssRNA virus species (38.03%), which were assigned to 20 virus families. In addition, 5 dsDNA virus species (1.12%) were assigned to 2 virus families, and 13 dsRNA virus species were assigned to 4 virus families. Three virus species were classified as unclassified (Figure 3A).

### 3.3. VIP-DB Platform

Finally, we developed VIP-DB to store and manage the collected data on viruses, plants, and insects. VIP-DB’s backend construction workflow is briefly as follows: various data (including literature, transmission relationships, and sequence information) are integrated and processed to generate a standardized Virus–Insect–Plant database for use by the front-end interface. The Virus–Insect–Plant DB platform is publicly accessible for free (available at: https://viplant.cn/#/, accessed on 14 June 2026). It mainly includes pages for Home, Browse, and Help, which are described briefly as follows.

Home: Provides an entry point for virus transmission mode queries, integrating links to authoritative databases such as PubMed, GPI, Virus–Host DB, and NCBI for quick access to relevant information. It also displays the distribution of virus and insect vector families. Browse: Supports comprehensive browsing and retrieval of data on viruses, host plants, and insect vectors. Help: Provides a detailed system guide covering the operation of the Home and Browse modules.

## 4. Discussion

Several existing databases and platforms provide important foundations for plant virus research, but they differ from VIP-DB in data scope and organization. GPI-base focuses mainly on *Geminiviridae*-related plant–insect relationships and is therefore highly useful for studies of geminiviruses, but it covers a narrower viral taxonomic range. The Plant Virus Transmissions Database provides broad information on plant virus transmission modes and vectors across a large number of plant viruses and publications, making it a valuable reference for transmission biology. However, VIP-DB was designed with a different emphasis: it organizes manually checked virus–insect transmission records together with host plant information, transmission mode annotation, taxonomic information, and literature evidence within a unified virus–insect–plant relationship framework. Therefore, VIP-DB should be regarded as a complementary resource rather than a replacement for existing databases. Its main contribution is to provide an evidence-traceable and queryable framework for documented virus–insect–plant relationships.

The results showed that most viruses were transmitted by only 1 insect species, and most insects transmitted only a single virus. For example, 45 insect species were found to transmit only 1 virus, and 378 viruses were transmitted by only 1 insect species, indicating that many documented virus–insect transmission relationships in the current dataset show a relatively high degree of specificity. In addition, the study also identified biologically significant cases where some viruses could be transmitted by multiple insect species, or some insects could transmit more than one virus. *Bemisia tabaci* transmitted 254 virus species, and *Myzus persicae* transmitted 39 virus species, indicating that certain insect vectors possess specialized transmission capabilities [7,19]. Meanwhile, TSWV, transmitted by 11 insect species, demonstrates the adaptive transmission capacity of a single virus. The number of virus species transmitted by *Bemisia tabaci* was significantly higher than that of other insect vectors [20,21].

Notably, *Hemiptera* insects are closely associated with the transmission of *Geminiviridae*, transmitting viruses from 28 families. This broad transmission pattern may be related to the specialized piercing-sucking mouthparts of *Hemiptera*, which facilitate virus delivery into plant tissues [22]. Among *Hemiptera*, *Bemisia tabaci* stands out as a particularly effective vector, transmitting the largest number of virus species and significantly outranking other insects, which highlights the variation among Hemiptera species in the number of plant virus species they transmit [23,24].

*Aphididae* is the most speciose family within the order *Hemiptera*. Common traits of *Aphididae* associated with their piercing-sucking feeding strategy explain their high efficiency in transmitting plant viruses, particularly noncirculative ones. Key traits include specialized maxillary stylets with a distal acrostyle that mediates viral particle binding, and frequent, brief (5–10 s) intracellular test probes on epidermal/mesophyll cells that enable rapid viral acquisition and inoculation [22]. Notably, *Aphididae* can transmit non-persistent viruses to plants without establishing sustained feeding [25]: this occurs via transient exploratory stylet punctures for plant cue sampling, where stylet-borne viruses are inoculated into healthy plants through salivary secretion or acquired from infected ones, with no need for phloem ingestion [26]. Furthermore, *Aphididae* exhibits altered feeding behaviors on non-host plants, such as more frequent test probing, shorter penetration durations, and no deep vascular tissue exploration. These changes enhance non-persistent virus transmission by increasing stylet–plant cell contact events (each a potential inoculation opportunity) and accelerating aphid dispersal to new hosts [27]. Thus, detailed studies on aphid feeding behavior biology, its modulation on host/non-host plants, and mechanistic links to virus transmission at the stylet–plant interface may provide novel insights into plant virus spread mechanisms.

Interestingly, plant virus transmission modes appeared to be associated with envelope presence in our dataset. Non-persistent transmission was not observed among enveloped viruses, whereas non-enveloped viruses exhibited a broader range of transmission modes. This pattern is consistent with previous reports indicating that enveloped plant viruses are generally transmitted in a persistent-propagative manner and that different transmission modes involve distinct virus–vector interaction mechanisms. However, further studies are needed to determine whether viral envelopes mechanistically constrain non-persistent transmission.

Despite the value of this study, several limitations remain. First, data bias can influence the interpretation of the results. Because the data were collected from the literature and public databases, well-studied viruses or insect vectors tend to be better represented, whereas less-studied relationships may have insufficient records. For example, some viruses recorded as being transmitted by only a single insect species, or some insects recorded as transmitting only one virus, may have additional undocumented transmission relationships due to limited identification efforts. Second, the high number of virus species associated with *Bemisia tabaci* in VIP-DB may reflect extensive research attention and the inclusion of GPI-base records, rather than only a true biological difference. Finally, the observed relationship between *Hemiptera* and *Geminiviridae* may represent only one well-documented part of a broader virus–insect interaction network, and other insect orders may also interact with viral families that remain insufficiently studied.

In summary, we developed VIP-DB, a curated virus–insect–plant database that provides researchers with an accessible platform for querying documented plant virus transmission records. The platform enables users to access plant virus, insect vector, host plant, transmission mode, taxonomic information, and supporting literature evidence. Future updates will incorporate newly published literature and additional public database records after manual evidence checking.

## Data Availability

The original contributions presented in this study are included in the article/Supplementary Material. Further inquiries can be directed to the corresponding authors.

## Supplementary Materials

The following supporting information can be downloaded at: https://www.mdpi.com/article/10.3390/v18060679/s1, Table S1. Virus–Insect–Plant relationships.
